# Epigenetic Biomarkers in Temporomandibular Joint Osteoarthritis: An Emerging Target in Treatment

**DOI:** 10.3390/ijms26083668

**Published:** 2025-04-12

**Authors:** Schilin Wen, Javiera Santander, Daniel Barria, Luis A. Salazar, Cristian Sandoval, Consuelo Arias, Verónica Iturriaga

**Affiliations:** 1 Grupo de Investigación de Pregrado en Odontología, Universidad Autónoma de Chile, Temuco 4811230, Chile; schilin.wen@cloud.uautonoma.cl (S.W.); santanderjaviera43@gmail.com (J.S.); daniel.barria1@cloud.uautonoma.cl (D.B.); 2 Sleep & Pain Research Group, Faculty of Dentistry, Universidad de La Frontera, Temuco 4811230, Chile; 3 Center of Molecular Biology and Pharmacogenetics, Scientific and Technological Bioresource Nucleus, Universidad de La Frontera, Temuco 4811230, Chile; luis.salazar@ufrontera.cl; 4 Escuela de Tecnología Médica, Facultad de Salud, Universidad Santo Tomás, Los Carreras 753, Osorno 5310431, Chile; 5 Departamento de Medicina Interna, Facultad de Medicina, Universidad de La Frontera, Temuco 4811230, Chile; 6 Escuela de Medicina, Facultad de Medicina y Ciencias de la Salud, Universidad Mayor, Santiago 8580745, Chile; consuelo.arias@gmail.com; 7 Department of Integral Adult Care Dentistry, Temporomandibular Disorder and Orofacial Pain Program, Universidad de La Frontera, Temuco 4811230, Chile

**Keywords:** osteoarthritis, temporomandibular joint, epigenetic, biomarkers

## Abstract

Osteoarthritis (OA) of the temporomandibular joint (TMJ) is a progressive disease characterized by the progressive destruction of the internal surfaces of the joint. Certain epigenetic biomarkers have been detected in TMJ-OA. We summarized the available evidence on the epigenetic biomarkers in TMJ-OA. There is an increase in the expression of non-coding RNAs related to the degradation of the extracellular matrix, chondrocyte apoptosis, and proinflammatory cytokines, while there is a decrease in the expression of those related to COL2A1, as well as the osteogenic and chondrogenic differentiation of mesenchymal stem cells. Certain methylated genes and histone modifications in TMJ-OA were also identified. In the early stage, DNA methylation was significantly decreased; that is, the expression of inflammation-related genes such as TNF and genes associated with extracellular matrix degradation, such as Adamts, were increased. While in the late stage, there was an increase in the expression of genes associated with the TGF-β and MAPK signaling pathway and angiogenesis-related genes. Although research on the role of epigenetic markers in TMJ-OA is still ongoing, the results here contribute to improving the basis for the identification of accurate diagnostic and prognostic markers and the development of new therapeutic molecules for the prevention and management of TMJ-OA. It also represents a significant advancement in elucidating its pathogenesis.

## 1. Introduction

### Osteoarthritis of the Temporomandibular Joint

Osteoarthritis (OA) of the temporomandibular joint (TMJ) is a chronic degenerative disease characterized by the progressive destruction of the internal surfaces of the joint and a reduction in the synovial fluid, causing the loss of joint function and a negative impact on the individual’s quality of life [1]. The clinical signs of OA include pain, grating, radiographic bone changes, and the narrowing of the joint space [2]. The main risk factors for OA are aging, joint trauma, and genetic predisposition [3], although emerging evidence suggests that a wider range of factors contribute to the development and progression of OA.

Physical therapy, non-steroidal anti-inflammatory drugs, arthroscopic procedures, and intra-articular injections of viscoelastic agents such as synthetic hyaluronic acid (HA) are commonly employed in managing TMJ-OA [4]. While these approaches may provide symptomatic relief, the limited regenerative potential of joint cartilage often prevents the full restoration of function or the reversal of cartilage damage, particularly in advanced stages of the condition [5]. In such cases, total joint replacement may become necessary if non-surgical interventions prove insufficient [6].

Although the pathology in TMJ-OA resembles that seen in the hip, knee, and hand joints, the TMJ has greater regenerative potential than hyaline cartilage-lined joints due to its fibrocartilaginous structure [7]. In contrast to hyaline cartilage, which primarily consists of type II collagen, the fibrocartilage of the TMJ contains both type I and type II collagen fibers. This formulation enhances the TMJ’s ability to endure shear forces and significant occlusal stresses encountered during mastication, hence augmenting its resistance and regenerative potential [8]. These differences have also been observed in inflammatory markers, which also depend on the type of cell affected and the type of mechanical stress to which they are subjected [9,10,11]. This has been observed in experiments despite inducing the same mechanical stress in fibroblasts; if these cells are derived from the knee joint, they increase IL-1 expression while those derived from TMJ tend to decrease its expression [12], suggesting that risk factors could have a different impact on TMJ compared to other joints with OA.

At present, the etiology and pathogenic mechanism of TMJ-OA continue to be unclear. In recent years, innovative advances in epigenetic markers related to OA have revealed their role in the pathogenesis of this disease and have been postulated as targets for therapeutic strategies [13,14,15,16,17]. Based on the differences that TMJ has with other joints, TMJ-OA deserves a specific review on epigenetic markers that could further clarify the pathogenesis and contribute to the development of new therapeutic targets.

OA represents the predominant type of arthritis and is a significant contributor to disability in the elderly population globally. This condition is a chronic, progressive joint disease marked by the degeneration of articular cartilage, synovial inflammation, and alterations in the subchondral bone. The global burden of OA is significant, with its increasing prevalence linked to aging populations and higher obesity rates. OA is a multifactorial condition influenced by various factors, such as occupational load, participation in high-impact sports, prior musculoskeletal injuries, obesity, and biological sex. These determinants influence both the onset of disease and its progression, as well as clinical outcomes. An in-depth comprehension of these risk factors is crucial for formulating specific prevention and management strategies. The recent literature emphasizes the necessity for a holistic approach to osteoarthritis that integrates both mechanical and systemic factors, highlighting its importance for public health [18,19].

OA is a multifaceted condition that presents varied clinical presentations based on its anatomical locations, natural development, clinical subgroups, and other etiological factors. The articular cartilage in a healthy joint can withstand significant stresses resulting from weight-bearing and joint movement over an individual’s lifetime. A hypothesis was established that chronic excessive stress and impaired biomechanical variables adversely affect the joint, resulting in the deterioration of articular cartilage and an ensuing inflammatory response. As a result, these symptoms subsequently led to stiffness, edema, and diminished mobility. Osteoarthritis is currently understood as a complex disease that involves several inflammatory and metabolic factors [20,21,22].

An increasing amount of research has highlighted the need to consider OA, including TMJ-OA, as a complex, multifaceted illness where genetic, mechanical, and environmental variables interact. Therefore, applying epigenetic research to the study of TMJ-OA has a lot of potential to clarify its pathophysiology and create more specialized and efficient therapies.

## 2. Epigenetics and OA

Epigenetics is described as the study of the changes in gene expression that occur regardless of the changes in the primary DNA sequence. Epigenetic phenomena are necessary in the control of gene expression, supporting correct cell development and function, and are also correlated with several diseases [23]. This has been demonstrated in monozygotic twins that share exactly the same genetic material but not necessarily the same epigenome [24]. Three main mechanisms are described as being involved in epigenetic regulation: (i) a post-translational histone modification that alters the chromatin conformation, (ii) non-coding RNAs (i.e., microRNAs, long non-coding RNAs, and circular RNAs), and (iii) DNA methylation [25,26].

**Figure 1:**
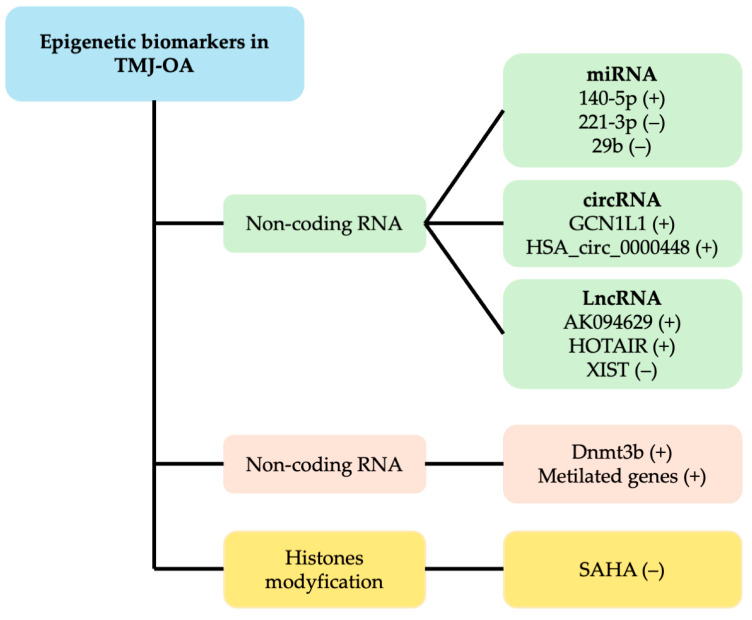
Summary of epigenetic biomarkers in TMJ-OA found in this review: (+) upregulated; (−) downregulated. Upregulated and downregulated non-coding RNAs are related to increased expression of genes that code for MMPs and proinflammatory cytokines. Significantly decreased DNA methylation, i.e., hypomethylated inflammation-related genes such as TNF and genes associated with ECM degradation, such as Adamts, was identified. Likewise, decreased Dnmt3b induces the appearance of TMJ-OA by downregulating type II collagen but upregulates Col X through Wnt/β-catenin activation. In contrast, an increased expression of miRNA221-3p, miRNA-29b, LncRNA XIST, and SAHA is related to osteogenic and chondrogenic differentiation of MSCs, COL2A1 expression, protective effects on cartilage, and decreased MMPs. Abbreviations: DNA: deoxyribonucleic acid, miRNA: micro-RNA, lncRNA: long non-coding RNA, circRNA: circular RNA, Dnmt3b: DNA methyltransferase 3b, SAHA: histone deacetylase inhibitor suberoylanilide hydroxamic acid, ECM: extracellular matrix, TNF: tumor necrosis factor, MMP: matrix metalloproteinase.

In recent years, growing attention has been directed toward the role of epigenetic changes in human OA, as demonstrated by the emergence of numerous genome-wide methylation studies [14]. These findings have led to the hypothesis that such epigenetic mechanisms may contribute to the development of bone-related conditions like OA. Consequently, we have compiled and reviewed the current data on epigenetic markers linked to TMJ-OA. A summary of epigenetic biomarkers in TMJ-OA are shown in Figure 1.

### 2.1. Post-Translational Histone Modification

Histone modifications, such as acetylation, methylation, phosphorylation, and ubiquitination at lysine residues within histone cores, represent key epigenetic markers that influence the ability of transcriptional machinery to access specific DNA regions [27,28].

Lysine acetylation, in particular, is a highly dynamic process controlled by two opposing enzyme families: histone acetyltransferases (HATs) and histone deacetylases (HDACs) [27]. HATs utilize acetyl coenzyme A (acetyl-CoA) as a substrate to transfer an acetyl group to the ε-amino group of lysine side chains. This neutralizes lysine’s positive charge, thereby reducing the affinity between histones and DNA [29].

Histone acetyltransferases are broadly divided into two categories: type A and type B. Type A HATs are primarily involved in acetylating histones within nucleosomes as well as other chromatin-related proteins, playing a direct role in transcriptional regulation. In contrast, type B HATs are located in the cytoplasm and acetylate newly synthesized histones, without directly impacting gene transcription [30].

Based on sequence homology and functional traits, HATs are further grouped into three major families: GNAT, MYST, and p300/CBP (Table 1), along with two types of co-activators—transcriptional co-activators and steroid receptor co-activators [31]. Type B HATs, which primarily reside in the cytoplasm, target free histones rather than those already integrated into chromatin structures [27]. This group is evolutionarily conserved and includes members like scHAT1, one of the earliest HATs identified. Type B HATs specifically acetylate newly formed histone H4 at lysine positions 5 and 12—common sites for post-translational modifications—as well as certain lysines on H3. These acetylation marks are crucial for proper histone deposition and are typically removed after incorporation into the chromatin [27,28].

HDACs, on the other hand, promote histone deacetylation, leading to increased chromatin condensation and the inhibition of gene transcription [38]. Histone deacetylases (HDACs) are enzymes critical for gene expression regulation through modifications of histone proteins. They function by removing acetyl groups from lysine residues on histones, leading to a more compact chromatin structure and subsequent transcriptional repression. HDACs are classified into four categories (I, II, III, and IV), with Classes I, II, and IV being zinc-dependent, while Class III (sirtuins) requires NAD+ as a cofactor. The role of HDACs extends to various biological processes, including cell cycle regulation, differentiation, and apoptosis. Abnormal HDAC activity is associated with multiple diseases, such as cancer, neurological disorders, and cardiovascular conditions. Consequently, HDAC inhibitors (HDACis) have emerged as potential therapeutic agents, showing promise in treating malignancies and other disorders [39].

Basically, HATs activate gene expression by loosening the chromatin, while HDACs repress it by tightening the chromatin structure; together, they maintain the balance of gene regulation through histone acetylation dynamics.

#### Histone Modification and TMJ-OA

Recent research indicates that HDAC inhibitors may offer therapeutic benefits in OA, although the specific mechanisms involved are not yet fully understood [40,41].

One such inhibitor, Trichostatin A (TSA), has been found to suppress the expression of matrix metalloproteinases (MMP-1, MMP-3, and MMP-13) induced by IL-1β stimulation. This suggests that inflammation in OA-affected chondrocytes may enhance HDAC expression and activity, potentially contributing to disease progression [42,43]. TSA has also demonstrated the ability to reduce synovial inflammation and protect cartilage from degradation in a murine model of collagen antibody-induced arthritis [41].

Additionally, TSA and another HDAC inhibitor, butyric acid, have been shown to inhibit IL-1β-triggered production of nitric oxide and prostaglandin E2 in human chondrocytes [44]. Similarly, vorinostat, another HDAC inhibitor, promotes anti-catabolic responses in chondrocytes by preventing NF-κB translocation to the nucleus [45]. Further in vitro studies have revealed that suppressing HDAC7 activity can reduce IL-1β-induced upregulation of MMP-13, indicating that HDAC7 may mediate the inflammatory response leading to MMP-13 expression in OA chondrocytes [46].

Sun et al. [32] evaluated the effects of suberoylanilide hydroxamic acid (SAHA), an HDACi. Synovial samples from eight patients with TMJ-OA who underwent TMJ surgery were used. In parallel, a rat model that was randomly assigned to an experimental group with TMJ-OA induced by collagenase I and a control group without OA was used. It was found that SAHA reduced IL-6 without affecting cell proliferation in SMSC and inhibited the activation of IL-1β-induced NF-κB.

Interleukin-1β is known to impair cartilage integrity by suppressing collagen production and promoting the release of matrix-degrading enzymes like MMP-13 in articular chondrocytes, leading to cartilage breakdown [47,48,49]. Additionally, IL-1β interferes with the immunomodulatory functions of mesenchymal stem cells (MSCs) and inhibits their ability to differentiate into chondrocytes within the joint environment [48,50].

SAHA promotes the expression of monocyte chemotactic protein-1-induced protein-1, a negative feedback regulator of IL-6, in human chondrocytes. This, in turn, helps suppress IL-6 production triggered by IL-1β stimulation [51].

SAHA also mitigated joint cartilage degradation induced by collagenase and further reduced the expression of inflammatory cytokines and proteases that were harmful to cartilage in the synovium. In addition to safeguarding chondrocytes and mitigating synovial inflammation, the HDAC inhibitors SAHA and LBH589 prevented IL-1β-induced IL-6 overexpression and enhanced the capacity of MSCs to develop into cartilage, which was suppressed by IL-1β [32].

SAHA might have protective effects on the cartilage, and MARK4 expression could be downregulated by SAHA in SMSC, which suggests that SAHA blocks the expression of IL-1β-induced IL-6 by inhibiting the MARK4/NF-κB pathway, a signaling pathway involved in the upregulation of cell inflammation [32,51].

In summary, HDAC inhibitors show therapeutic potential in OA by modulating inflammatory responses and protecting cartilage. Trichostatin A and butyric acid reduce IL-1β-induced expression of matrix-degrading enzymes and inflammatory mediators in chondrocytes. Vorinostat and HDAC7 inhibition also demonstrate anti-catabolic effects by blocking NF-κB activity. SAHA, another HDAC inhibitor, decreases IL-6 expression and synovial inflammation in both human and animal OA models. SAHA’s protective effects are linked to the inhibition of the MARK4/NF-κB pathway, enhancing cartilage regeneration capacity.

### 2.2. Non-Coding RNAs

#### 2.2.1. MiRNAs

miRNAs are short, non-coding RNA molecules, typically ranging from 20 to 24 nucleotides in length, that regulate gene expression post-transcriptionally. They accomplish this by binding to the 3′ untranslated regions (3′ UTRs) of target messenger RNAs (mRNAs), leading to mRNA degradation or the inhibition of translation [52,53].

Recent progress has greatly expanded our knowledge on the miRNA biogenesis pathway [52,54]. In animals, miRNAs are initially transcribed by RNA polymerase II as long primary transcripts known as pri-miRNAs [55,56]. These pri-miRNAs are then processed in the nucleus by the RNase III enzyme Drosha into precursor miRNAs (pre-miRNAs) with a characteristic hairpin structure [57]. Pre-miRNAs are transported from the nucleus to the cytoplasm through the action of exportin-5 (Exp5) [58,59,60], where they are further cleaved by the RNase III enzyme Dicer into double-stranded miRNA duplexes that are approximately 22 nucleotides in length [61,62,63,64,65]. From this duplex, one strand is typically selected to function as the mature, active miRNA [66,67].

In general, miRNAs are generated from large primary precursors (pri-miRNAs) transcribed either from introns of protein-coding genes or long non-coding RNA genes or from intergenic regions of the genome [68]. They have essential functions in the control of the gene expression of the processes of cell differentiation, proliferation, and apoptosis [69].

#### 2.2.2. MiRNAs and TMJ-OA

It has been mentioned that the expression of some microRNAs in normal human articular cartilage and osteoarthritic cartilage is different [33,70]; therefore, it is suggested that several miRNAs have been linked to the pathogenesis of osteoarthritis.

miR-146a has been recognized as an important modulator of inflammation, as it directly targets key upstream components of the NF-κB signaling pathway, specifically IL-1 receptor-associated kinase 1 (IRAK-1) and TNF receptor-associated factor 6 (TRAF-6) [71]. Moreover, elevated expression of miR-146a in chondrocytes has been shown to suppress the IL-1β-induced release of TNF-α [72]. These observations indicate that miR-146a holds promise both as a biomarker for osteoarthritis and as a potential therapeutic target for controlling inflammatory and catabolic responses in chondrocytes and synoviocytes.

miR-140 is known to be predominantly expressed in cartilage and has been extensively studied in this context. Research has shown that miR-140 levels are decreased in the articular cartilage of individuals with osteoarthritis. In mouse models, the absence of miR-140 leads to a mild skeletal growth defect and, more notably, increases susceptibility to osteoarthritis in adult cartilage, particularly following aging or surgical destabilization of the knee joint. This postnatal cartilage degeneration may be linked to miR-140’s role in downregulating catabolic enzymes such as MMP-13 and ADAMTS-5, which are upregulated by IL-1β [73,74,75].

Another microRNA implicated in OA is miRNA-127-5p. Its expression is upregulated in OA, leading to enhanced production of MMP-13 in human chondrocytes [13]. MMP-13 is a key enzyme responsible for degrading the extracellular matrix (ECM) in OA cartilage, particularly targeting type II collagen with high efficiency. As a pivotal factor in cartilage breakdown and disease progression, MMP-13 has emerged as a promising target for therapeutic interventions [76].

There are also miRNAs that are downregulated in OA. This is the case for miR-214-3p in chondrocytes from human and mouse samples in an experimental study. The treatment of chondrocytes with IL-1β decreased the expression of miR-214-3p, as it significantly reduced the damaged regions compared to the undamaged regions of osteoarthritic cartilage. In undamaged cartilage regions, miR-214-3p localized to the chondrocyte cytoplasm. The downregulation of miR-214-3p promotes ECM degradation and chondrocyte apoptosis through the activation of the NF-κB pathway because miR-214-3p significantly suppresses the activation of this pathway via IL-1β stimulation [77].

In the context of the TMJ, alterations in certain miRNA expression levels have also been identified. Li et al. [78] used mandibular condylar cartilage (MCC) from mice exposed to IL-1β to create an in vitro model of TMJ-OA. Their results showed elevated levels of MMP-13, miR-140-5p, and NF-κB in treated samples compared to healthy controls. Additionally, miR-140-5p was found to directly suppress Smad3, a molecule that plays a role in downregulating TGF-β. These irregularities in miR-140-5p expression may signal the onset of TMJ-OA. Thus, miR-140-5p could influence TMJ-OA development via the modulation of the TGF-β/Smad pathway and may serve as an early biomarker for the disease [78].

These molecular alterations are not limited to cartilage but also occur in the synovial membrane fibroblasts of the TMJ. Xu et al. [70] analyzed human synovial fibroblasts from individuals with TMJ-OA and discovered a fivefold reduction in miRNA-221-3p expression compared to healthy controls. This downregulation was associated with a significant upregulation of MMP-1 and MMP-9. Furthermore, the expression of the transcription factor Ets-1 was elevated in TMJ-OA fibroblasts. Silencing the gene encoding Ets-1 reversed the enhanced expression of MMP-1 and MMP-9, indicating that miRNA-221-3p suppresses these matrix metalloproteinases by directly targeting Ets-1. As a key member of the Ets transcription factor family, Ets-1 is found in various cell types including fibroblasts, B cells, endothelial cells, and neoplastic cells, and it has also been detected in TMJ fibrocartilage. Ets-1 plays an essential role in joint inflammation, particularly under hypoxic conditions and in the presence of proinflammatory stimuli. Therefore, miRNA-221-3p may modulate the course of TMJ-OA by regulating Ets-1 in synovial fibroblasts [79].

Regarding mesenchymal stem cells derived from the bone marrow of TMJ condyles affected by osteoarthritis, Sun et al. [32] investigated bone marrow mesenchymal stem cells (BMSCs) from condylar samples in a TMJ-OA model induced by unilateral anterior crossbite (UAC). They observed decreased expression of miRNA-29b alongside elevated levels of Wnt-5a, a member of the Wingless-type protein family, which coincided with subchondral bone loss in the condyle. Notably, targeted overexpression of miR-29b suppressed subchondral bone degradation, cartilage breakdown, and osteoclast overactivity. In contrast, miR-29b inhibition led to the worsening of these pathological features. Furthermore, increasing miR-29b expression resulted in a reduction in Wnt-5a levels, whereas miR-29b knockdown had the opposite effect. These findings suggest that miR-29b plays a central role in regulating the osteoclast-promoting activity of BMSCs and contributes to subchondral bone loss in TMJ-OA [80].

Therefore, several miRNAs are implicated in the development and progression of OA. miR-146a and miR-140 help regulate inflammatory pathways and cartilage integrity, while miR-127-5p and miR-214-3p influence cartilage degradation and cell death. In TMJ-OA, altered expression of miR-140-5p, miR-221-3p, and miR-29b affects inflammatory processes, matrix degradation, and bone loss. These miRNAs act through key molecular targets such as NF-κB, MMPs, Smad3, and Wnt-5a. Overall, they hold promise as biomarkers and therapeutic targets for OA and TMJ-OA.

#### 2.2.3. Long Non-Coding RNAs

Long non-coding RNAs (lncRNAs) are RNA transcripts longer than 200 nucleotides that lack protein-coding potential [32,81]. These molecules are capable of interacting with DNA, RNA, and proteins to influence various cellular processes.

Recent research highlights a strong association between lncRNA dysregulation and the pathogenesis of osteoarthritis (OA), particularly in knee OA models [82,83,84].

A common mechanism involves lncRNAs functioning as competitive endogenous RNAs (ceRNAs), where they bind to miRNAs, thereby preventing these miRNAs from suppressing their target genes. This regulatory interaction can influence key processes such as cell proliferation, programmed cell death, autophagy, and the degradation of the extracellular matrix (ECM), all of which are central to OA progression.

By modulating these pathways, lncRNAs may help control inflammation and support cartilage repair and homeostasis [82,85].

Moreover, certain lncRNAs serve as miRNA sponges and are involved in the regulation of MMP expression, affecting the progression of OA [84,85].

#### 2.2.4. LncRNA and OA

For instance, the overexpression of lncRNA-CIR and MMP-13 and low expression of miR-27b were found in OA patients, suggesting that there is a close relationship between them. miR-27b binds directly to lncRNA-CIR and MMP-13 [84].

#### 2.2.5. LncRNA and TMJ-OA

Synovium-derived mesenchymal stem cells (SMSCs) possess the capacity to differentiate into chondrocytes, making them promising candidates for articular cartilage regeneration in TMJ-OA [86,87]. Jia et al. [34] analyzed synovial tissue from ten TMJ-OA patients who underwent surgery and found that the lncRNA AK094629 was positively associated with IL-1β expression. Furthermore, IL-1β was shown to induce the upregulation of AK094629 in SMSCs cultured in vitro. AK094629 was also found to suppress MAP3K4, a kinase whose expression is otherwise enhanced by IL-1β. Interestingly, reducing MAP3K4 levels reversed IL-6 upregulation but did not alter AK094629 levels, indicating a downstream role in inflammatory signaling [34].

Another lncRNA, HOX transcript antisense RNA (HOTAIR), is markedly elevated in the synovial fluid of TMJ-OA patients when compared to healthy controls. Multiple studies have identified HOTAIR as a key player in OA pathology. It facilitates IL-1β-induced expression of matrix metalloproteinases (MMPs) [88] and promotes OA progression via the miR-17-5p/FUT2/β-catenin signaling axis [73]. HOTAIR has also been implicated in chondrocyte apoptosis regulation through an interaction with another lncRNA, PACER [89]. Additionally, osteopontin, a multifunctional phosphoprotein secreted by cells such as chondrocytes and osteoblasts, may drive HOTAIR expression. Osteopontin is overexpressed in OA and contributes to matrix degradation by inducing MMP-13, which targets type II collagen, a primary component of cartilage [90].

In rabbit models of TMJ-OA, HOTAIR levels were significantly increased in synovial fluid. Experiments using IL-1β-stimulated rabbit condylar chondrocytes showed a substantial rise in MMP-1, MMP-3, MMP-9, and HOTAIR expression. Notably, HOTAIR expression spiked early during IL-1β exposure and later returned to baseline levels despite ongoing stimulation, suggesting that HOTAIR acts as an early responder potentially influenced by additional signaling factors. Silencing HOTAIR reversed IL-1β-induced increases in MMPs and reduced apoptosis in chondrocytes, supporting its involvement in cartilage degeneration and TMJ-OA progression through MMP activation and apoptosis promotion [88].

Plasmacytoma variant translocation 1 (PVT1), an lncRNA with tumorigenic potential, also plays a role in TMJ-OA. PVT1 acts as a molecular sponge for miRNAs, preventing them from degrading target mRNAs. Specifically, PVT1 has binding affinity for miR-211-3p and can inhibit its regulatory effect. In vitro studies revealed that PVT1 is involved in chondrocyte apoptosis induced by synovial membrane inflammation. Synoviocytes stimulated with lipopolysaccharide exacerbated chondrocyte apoptosis; however, silencing PVT1 partially mitigated this effect. This suggests that PVT1 sequesters miR-211-3p, thereby lifting its suppression of TNF-α, which promotes chondrocyte apoptosis in TMJ-OA [91].

The lncRNA X-inactive specific transcript (XIST), which is located on chromosome Xq13.2, has been implicated in the development of TMJ-OA [92,93]. XIST is known to regulate key cellular functions such as proliferation, apoptosis, and differentiation across various cell types. Although previous studies have indicated that XIST suppresses osteogenic differentiation in MSCs, its influence on the chondrogenic potential of SMSCs has not been fully elucidated [94].

Zhu et al. [35] analyzed synovial tissue obtained from TMJ-OA patients undergoing surgical procedures and observed that the downregulation of XIST facilitated chondrogenic differentiation in SMSCs, as evidenced by increased expression of COL2A1. Mechanistically, XIST was shown to act as a molecular sponge for miR-27b-3p, a microRNA that negatively regulates A disintegrin and metalloproteinase with thrombospondin motifs 5 (ADAMTS-5), a key enzyme contributing to aggrecan degradation in OA [36,95]. The suppression of XIST resulted in a notable rise in miR-27b-3p levels compared to controls, suggesting that XIST modulates ADAMTS-5 expression by competitively binding to miR-27b-3p, thereby influencing ECM breakdown and cartilage degeneration [95].

SMSCs can differentiate into chondrocytes and may help repair cartilage in TMJ-OA. Several lncRNAs, including AK094629, HOTAIR, PVT1, and XIST, are involved in TMJ-OA pathogenesis. AK094629 and HOTAIR are upregulated by IL-1β and contribute to inflammation, MMP expression, and chondrocyte apoptosis. PVT1 promotes apoptosis via an interaction with miR-211-3p and TNF-α regulation. XIST inhibits chondrogenesis by sponging miR-27b-3p, thereby enhancing ADAMTS-5 expression.

#### 2.2.6. Circular RNAs (circRNAs)

circRNAs are a class of non-coding RNAs characterized by their covalently closed-loop structure, which is formed by the joining of their 3′ and 5′ ends [96]. Recent findings suggest that circRNAs act as natural microRNA (miRNA) sponges, binding to and inhibiting miRNA activity through competitive interactions. Despite these insights, the specific functions of circRNAs in cartilage tissue and their broader involvement in the pathogenesis of osteoarthritis (OA) remain largely undefined [36,96,97,98].

#### 2.2.7. circRNAs and OA

Multiple studies have identified altered circRNA expression profiles in osteoarthritic cartilage compared to healthy tissue, highlighting their potential roles in regulating chondrocyte function. For instance, circRNA-100876 has been shown to contribute to extracellular matrix (ECM) degradation by acting as a molecular sponge for miRNA-136. Similarly, has_circ_0005105 is notably upregulated in chondrocytes stimulated with IL-1β, where it exacerbates OA progression by competitively binding to miRNA-26a, ultimately promoting ECM breakdown [97].

#### 2.2.8. circRNA and TMJ-OA

circPDE4D, a circular RNA derived from the linear *PDE4D* gene, plays a vital role in preserving the ECM during the progression of OA. Notably, circPDE4D expression is markedly reduced in OA cartilage and in response to inflammatory cytokine exposure. Experimental silencing of circPDE4D led to a significant decrease in aggrecan levels and an increase in matrix-degrading enzymes, including MMP-3, MMP-13, ADAMTS-4, and ADAMTS-5, although it did not appear to influence cell proliferation or apoptosis [98].

FGF18, an important member of the FGF family, is recognized for its essential role in skeletal development and its therapeutic potential in cartilage repair for OA patients [99]. It promotes ECM synthesis and chondrocyte proliferation in vitro [100]. Interestingly, FGF18 is directly targeted by miR-103a-3p, whose activity is regulated by circPDE4D. Restoration experiments demonstrated that either overexpressing FGF18 or inhibiting miR-103a-3p could counteract the ECM-degrading effects triggered by circPDE4D knockdown, including the suppression of aggrecan and the induction of matrix-degrading enzymes. These findings suggest that circPDE4D modulates OA progression by sponging miR-103a-3p, thereby controlling FGF18 expression and contributing to ECM homeostasis [100].

#### 2.2.9. TMJ-OA and circRNA

Hu et al. [5] analyzed synovial tissue samples from ten individuals who had undergone TMJ articular disk procedures. The test group included patients diagnosed with TMJ-OA, while the control group consisted of individuals with anterior disk displacement but without TMJ-OA. They found that several circRNAs were upregulated, most notably hsa_circ_0000448, which appeared to be significantly involved in myogenesis and the TNF-α and IFN-γ signaling pathways. Bioinformatic analyses suggested that hsa_circ_0000448 may function as a miRNA sponge, potentially influencing TNF-α expression at both transcriptional and post-transcriptional stages. This could enhance TNF-α secretion in the synovial lining and contribute to the development of TMJ-OA. Conversely, the downregulated circRNAs were mainly associated with the activation of Wnt pathway receptors [5].

Zhu et al. [101] demonstrated that circRNA GCN1L1 was also differentially expressed in the synovial membrane of TMJ-OA samples induced experimentally in a rat model. This circRNA might be involved in TMJ synovitis and also demonstrated that it regulates miRNA-330-3p, which goes to TNF-α. Therefore, circRNA GCN1L1 would serve as a sponge for miRNA-330-3p, promoting the expression of TNF-α, an inflammatory cytokine key in the pathogenesis of TMJ-OA. This in vitro and in vivo experiment also confirms that circRNA GCN1L1 promotes the proliferation of synoviocytes and the apoptosis of chondrocytes through miR-330-3p and TNF-α. MiR-330-3p goes directly to the TNF gene and regulates the expression and secretion of TNF-α, and circRNA GCN1L1 induces the proliferation of synoviocytes, the apoptosis of chondrocytes, and the degradation of the cartilage ECM [101].

circPDE4D, a circular RNA derived from PDE4D, plays a crucial role in maintaining the ECM during OA progression. It is downregulated in OA cartilage and inflammation. Its knockdown leads to aggrecan loss and increased catabolic enzymes but does not affect proliferation or apoptosis. FGF18, a key cartilage regenerative factor, is regulated by circPDE4D through miR-103a-3p. circPDE4D acts as a sponge for miR-103a-3p, indirectly controlling FGF18 and ECM degradation in OA.

### 2.3. DNA Methylation

DNA methylation is one of the most extensively researched forms of epigenetic modification. It directly affects the DNA sequence by adding a methyl group to the fifth carbon of cytosine, resulting in gene silencing. This methylation can influence gene expression by blocking transcription factor binding sites, interfering with the transcriptional machinery, or interacting with methyl-CpG-binding domain proteins [102]. The process is regulated at multiple levels and is carried out by DNA methyltransferases (DNMTs), a group of enzymes that includes DNMT1, DNMT3A, and DNMT3B. These enzymes are vital for both establishing and maintaining DNA methylation patterns, playing a crucial role in development and homeostasis.

#### 2.3.1. DNA Methylation and OA

It has been described that aberrant DNA methylation causes multiple diseases, including OA. A study with samples of rat mandibular condyle cartilage with TMJ-OA induced experimentally in early, intermediate, and advanced stages of the disease was carried out by comparing them with healthy controls. It was observed that the pathways and differentially methylated genes changed dynamically with the progression of TMJ-OA, suggesting that epigenetic events play an important role in the progression of the disease [25,102].

Research has shown that different subtypes of OA patients can be identified based on distinct DNA methylation profiles [103,104], indicating that DNMTs may have a crucial function in the development of OA. Regarding inflammatory processes, an NF-κB binding site has been discovered in the promoter region of the murine DNMT3B gene, which is also conserved in the human version. Notably, human primary chondrocytes, either derived from OA patients or exposed to IL-1β, exhibit reduced DNMT3B expression. Additionally, when fibroblast-like synoviocytes are treated with IL-1β or TNF-α, there is a notable decrease in both the expression and activity of DNMT3A [52].

The methylation of DNA and the methylation changes to cytosine–guanine dinucleotides (CpGs), are more closely linked to the repression of gene expression of transcription factors such as the nuclear factor of activated T-cell transcription factor 1 (NFATc1) and Sry-related HMG box9 (SOX9); cytokines genes such as interleukin1-β (IL-1β) and tumor necrosis factor-α (TNF-α); collagen type II alpha 1 chain (COL2A1); aggrecan; and matrix-degrading proteinases (MMP-13). Studies analyzing DNA methylation have focused on identifying epigenetic marks and profiles that are associated with OA in bone and cartilage tissue [13].

#### 2.3.2. DNA Methylation and TMJ-OA

In the case of TMJ-OA, changes associated with DNA methylation have also been detected [25]. In fact, methylome studies revealed differential DNA methylation signatures in OA patients, indicating that such epigenetic-regulated changes in the DNA structure could be an important factor in OA development and progression [26,52,105,106,107]. In this sense, it has been reported that the methylation of certain genes that encode enzymes involved in the metabolism of articular cartilage could have a beneficial effect on TMJ-OA [108].

Comparative analyses of DNA methylation patterns in early-stage TMJ-OA and healthy controls identified 2198 genes with altered methylation, whereby 1681 of them were hypomethylated and 517 were hypermethylated. GO and KEGG pathway analyses revealed significant enrichment in biological processes tied to early disease activity, particularly those governing cell cycle regulation, immune responses, and intracellular signaling. Genes involved in inflammation and immune modulation, such as TNF, TNRSF1A, TRAF2, IL-7, IL-2RA, IL-4RA, IL-9RA, and IL-27RA, as well as those implicated in ECM breakdown, like ADAMTS5, ADAMTSL5, and ADAM1A, displayed pronounced hypomethylation [20].

At the late stage of TMJ-OA, researchers identified 3960 differentially methylated genes, of which 3061 were hypomethylated and 899 were hypermethylated. Notably, about 60% of these changes were exclusive to the late stage. While some genes related to ECM degradation (e.g., ECM1, COL1A2, COL27A1, COL4A4, and PRG4) and immune responses (e.g., Adamts and Ils) were already present in the early phase, new functional clusters emerged in later stages. These included the TGF-β signaling pathway (TGFB1, TGFB2, TGFBR2, ACVR2B, SMAD4, and SMAD7), the MAPK pathway (MAPK1, MAP2K4, MAP4K2, MAP3K7, and MAP3K13), angiogenesis and vascular development (VEGFA, CTGF, FGFR1, FGF1, PDGFA, PDGFB, ANGPTL4, and AKT3), and ossification regulation (OMD and MEPE) [25,107].

Members of the TGF-β superfamily play central roles in chondrocyte differentiation and maturation. For instance, TGF-β drives chondrogenesis and matrix production in developing limb structures but also suppresses chondrocyte hypertrophy and type X collagen expression during bone elongation. The disruption of TGF-β signaling in the growth plate results in a thicker hypertrophic zone and elevated Col X expression [109].

In a study by Zhou et al. [37], both chemically and surgically induced TMJ-OA models in rats and rabbits were employed, along with tamoxifen-induced Dnmt3b knockouts in chondrocytes. They observed that DNMT3B expression varied during condylar cartilage formation and declined as TMJ-OA progressed. Dnmt3b was shown to play a vital role in OA pathogenesis and, when overexpressed, could partially mitigate disease progression using stem/progenitor cells and in vivo systems. The expression of DNMT3B significantly decreased at 4 and 8 weeks after OA induction. The loss of DNMT3B led to enhanced chondrocyte proliferation and early hypertrophy of TMJ progenitor cells, contributing to disease initiation. This highlights DNMT3B’s role in maintaining chondrocyte stability and preventing pathological differentiation.

Moreover, for the first time, DNMT3B was identified as a potential therapeutic target. The overexpression of DNMT3B elevated type II collagen expression and reduced type X collagen expression, whereas its downregulation triggered TMJ-OA onset via reduced type II collagen expression and increased Col X expression, which involved the activation of the Wnt/β-catenin pathway [37,110]. Since Wnt/β-catenin signaling is pivotal for cartilage integrity and is linked to OA development [111,112,113,114], the study proposed an interaction between DNMT3B and this pathway. The attenuation of DNMT3B promoted β-catenin nuclear translocation both in vitro and in vivo, suggesting a mechanistic link. Further research is necessary to fully understand how DNA methylation affects Wnt/β-catenin-related genes in TMJ-OA.

**Figure 2:**
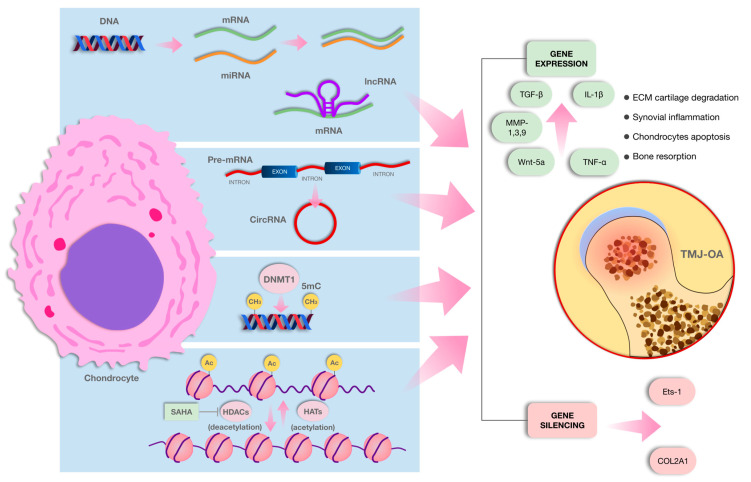
Schematic representation of the main epigenetic mechanisms described in this review. The figure includes the reported consequences of each mechanism in the extracellular matrix of the cartilage. Abbreviations: DNA: deoxyribonucleic acid, mRNA: messenger ribonucleic acid, miRNA: micro RNA, lncRNA: long non-coding RNA, circRNA: circular RNA, DNMT1: DNA methyltransferase 1, CH_3_: methyl group, Ac: acetylation, SAHA: histone deacetylase inhibitor suberoylanilide hydroxamic acid, HDACs: histone deacetylases, HATs: histone acetyltransferases, ECM: extracellular matrix, TNF-α: tumor necrosis factor alpha, TGF-β: transforming growth factor-beta, MMP: matrix metalloproteinase, WNT-5a: Wingless-type protein family member 5A, IL-1β: interleukin-1 beta, COL2A1: collagen, type II, alpha 1.

In TMJ-OA, DNA methylation changes play a key role in its development and progression, with differential methylation patterns identified in both early and late stages. Studies show that genes related to cartilage metabolism, immune responses, and ECM degradation are differentially methylated, with significant alterations in the TGF-β and MAPK signaling pathways at later stages. DNMT3B, a crucial regulator, is downregulated during OA progression, and its deficiency promotes disease onset by disrupting chondrocyte homeostasis. Furthermore, DNMT3B interacts with Wnt/β-catenin signaling, offering potential therapeutic insights for TMJ-OA. A schematic representation of the main epigenetic mechanisms described in this review are shown in Figure 2. Also, a summary of the methodological quality of the included studies is shown in Table S1. 

### 2.4. Knee/Hip OA

New studies show that TMJ-OA and knee/hip OA have different epigenetic landscapes. Differential gene regulation results from the local microenvironment and mechanical stimuli have been identified, even though both types entail epigenetic processes such DNA methylation, histone modification, and non-coding RNAs (miRNAs).

For example, TMJ-OA has been linked to the differential methylation of genes that are implicated in inflammation and cartilage homeostasis (e.g., MMP13, COL2A1, and ACAN) [115,116]. Research indicates that TMJ chondrocytes exhibit dysregulation of miR-140 and miR-146a, which affects matrix breakdown enzymes and inflammatory cytokines [117,118].

On the other hand, knee OA typically entails increased catabolic pathways and wider epigenetic suppression of anabolic genes throughout weight-bearing cartilage zones. For instance, cartilage deterioration in knee OA has been connected to alterations in DNA methylation in the promoters of RUNX2 and SOX9 [119,120].

Furthermore, by affecting mechanoresponsive gene expression and chromatin remodeling, mechanical loading patterns, which vary greatly between the TMJ and major joints like the knee or hip, likely shape these epigenetic markers. A summary of the features of TMJ-OA and knee/hip OA is shown in Table 2.

### 2.5. Scope and Limitations

Despite significant advancements in understanding the role of epigenetic modifications in TMJ-OA, several limitations remain. A major challenge is the limited availability of clinical studies, as most findings are derived from preclinical and in vitro models, necessitating further validation in human cohorts. Additionally, the heterogeneity of study methodologies, including differences in sample sources and experimental conditions, complicates direct comparisons and reproducibility. Another critical issue is the lack of longitudinal data, making it difficult to assess how epigenetic changes evolve over time and their precise role in disease progression. Furthermore, the intricate interactions between non-coding RNAs, DNA methylation, and histone modifications create a complex regulatory network, making it challenging to isolate the specific contributions of individual epigenetic alterations.

While epigenetic biomarkers hold promise for diagnosis and treatment, their translation into therapeutic applications faces multiple hurdles, including challenges related to targeted delivery, potential off-target effects, and long-term safety concerns. The specificity of TMJ-OA also requires deeper investigation, as most epigenetic research has focused on larger weight-bearing joints such as the knee and hip. Understanding TMJ-specific molecular mechanisms is essential for developing effective, tailored interventions. Addressing these limitations through well-designed, large-scale clinical studies and standardized methodologies will be crucial for advancing epigenetic research in TMJ-OA.

### 2.6. Future Studies

To bridge the gap between epigenetic discoveries and clinical applications, future research should prioritize the validation of identified biomarkers in large patient cohorts to assess their diagnostic and prognostic potential. Additionally, therapeutic exploration should focus on developing and optimizing HDAC inhibitors, miRNA mimics, and DNA methylation modulators as potential treatment strategies. Integrating multi-omics approaches by combining epigenomics, transcriptomics, and proteomics could provide a more comprehensive understanding of TMJ-OA pathogenesis and help uncover novel regulatory networks involved in disease progression.

The creation of efficient and secure delivery methods, particularly those capable of targeting specific tissues such as cartilage or synovial fluid, is essential to ensure clinical relevance and minimize off-target effects. Furthermore, advancements in gene-editing technologies such as CRISPR/Cas9 present significant potential for facilitating precise epigenetic alterations in relation to TMJ-OA. Extensive cohort studies utilizing comprehensive methylome analysis throughout different stages of TMJ-OA are essential to enhance our understanding of the role of epigenetic alterations in disease development and progression. This research may reveal the dynamic nature of epigenetic alterations throughout time, especially if conducted longitudinally. Simultaneously, preclinical studies ought to assess the efficacy of epigenetic modulators, including HDAC inhibitors (e.g., vorinostat and panobinostat), which may restore the control of genes associated with inflammation and cartilage repair. Innovative technologies like vibroacoustic analysis offer non-invasive techniques for detecting subtle changes in joint function, which may indicate early epigenetic dysregulation. The integration of functional diagnostics with epigenetic biomarkers may facilitate early detection and enhanced the monitoring of the progression of TMJ-OA. Moreover, further investigation is necessary to evaluate the alteration of significant miRNAs associated with inflammation and cartilage degradation; yet miRNA-based therapies present a compelling alternative. The utilization of personalized medicine approaches, supported by multi-omics data, has the potential to enhance therapeutic efficacy by tailoring treatments for specific patient subgroups, considering the considerable diversity in individual epigenetic profiles. Ultimately, further clinical research focusing on epigenetic dynamics and environmental interactions is necessary to substantiate these methodologies and transform them into effective diagnostic and therapeutic modalities for TMJ-OA.

## 3. Conclusions

Epigenetic modifications play a crucial role in the pathogenesis of TMJ-OA by regulating gene expression related to inflammation, extracellular matrix degradation, and chondrocyte apoptosis. Non-coding RNAs, DNA methylation, and histone modifications collectively contribute to disease progression and present novel opportunities for biomarker identification and targeted therapies. However, despite promising findings, the translation of epigenetic discoveries into clinical applications is still in its early stages. Understanding TMJ-specific epigenetic patterns will be essential for developing personalized treatment approaches.

One of the main problems still unresolved in OA is the impossibility of repairing the articular cartilage, especially in advanced stages. The study of molecular markers has contributed to the knowledge of the pathogenesis of OA and therefore to gene and epigenetic therapy. In recent years, epigenetics has received increasing attention due to its involvement in a variety of biological processes and diseases. non-coding RNAs, DNA methylation, and histone protein modifications constitute a group of genome modifications that influence gene expression without altering the DNA sequence. This software is called epigenome, and it is malleable, and its composition can be influenced by environmental factors. We found that inflammation-induced epigenetic changes may play an important role in ECM imbalance, TMJ-OA pathogenesis, and disease progression. We assume that genetic engineering, gene editing, the use of gene-targeting methods such as ZFN, TALE, and CRISPR/Cas9 will be an innovative research field in the area of TMJ-OA.

According to this review, there is an increase in research on epigenetics in OA; however, articles on TMJ-OA are still few and recent. The study of the main signaling pathways involved in inflammation and the degradation of the cartilage ECM is key to identifying epigenetic biomarkers in TMJ-OA. In this sense, the TMJ has structural and functional characteristics that are different from other joints. TMJ fibrocartilage differs biologically and histologically from hyaline cartilage, which deserves to be treated especially. Non-coding RNAs can form a very complex network, where LncRNAs and circRNAs interact with various miRNAs, and one miRNA could, in turn, inhibit various target genes and act as sponges. Furthermore, it has been observed that DNA methylation profiles are different depending on the severity of the TMJ-OA.

Challenges and implications in TMJ-OA include non-coding RNA-targeted therapies related to increased expression of genes involved in cartilage regeneration and chondrocyte protection. Clinical studies, focused on preventing progression and reversing damage, in patients with TMJ-OA are also needed to test the efficacy of epigenetic therapies at different stages of the disease. The presence of stable epigenetic modifications in cells indicates potential imprinting, offering valuable insights into disease mechanisms and the identification of critical pathways involved in inflammation and extracellular matrix degradation.

Ultimately, one of the major challenges of implementing gene therapy lies in its high cost. Although joint disorders are widespread, frequently chronic, and can cause significant disability, their non-lethal nature brings into question whether the therapeutic benefits justify the associated risks and expenses.

## Figures and Tables

**Table 1:** Characteristics and results of epigenetic biomarkers in TMJ-OA.

Reference	TMJ Sample	Sample Characteristics	Epigenetic Biomarkers	Main Results
[5]	Synovial tissues	TMJ-OA patients undergoing TMJ disk surgery.	circRNA	circRNA_0000448 upregulation is related to the TNF, IL-1, and IFN-signaling pathways, and downregulated circRNAs are related to myogenesis.
[25]	Articular cartilage	Surgically induced TMJ-OA rats.	DNA methylation	There was significant differential methylation of several genes involved in the pathogenesis of TMJ-OA. In the early stage, this resulted in the methylation of genes of the TNF family, such as Adamts5 and Runx. In late stage, this included genes of the VEGFA, CTGF, MEPE, and OMD families.
[32]	SMSCs	Patients with TMJ-OA undergoing TMJ debridement surgery.	Histone deacetylation	SAHA attenuated IL-6 secretion in IL-1β-induced SMSCs through the inhibition of the MARK4/NF-κB pathway.
[32]	BMSCs of subchondral bones	A mouse model with an OA-like change in the TMJ induced by an experimentally UAC.	miRNA	miR-29b was markedly lower in BMSCs from subchondral bones of TMJ-OA.
[33]	Synovial tissues	TMJ-OA patients	miRNA	IL-1β reduced miRNA221-3p expression in a time- and dose-dependent manner in TMJOA synovial fibroblasts. The expression of Ets-1 was induced.
[34]	SMSCs	Patients with TMJ-OA were treated surgically.	lncRNA	The downregulation of lncRNA AK094629 attenuated IL-1β-regulated IL-6 expression in TMJ -OA SMSCs by inhibiting MAP3K4.
[35]	SMSCs	Patients with TMJ-OA under- going TMJ surgery.	lncRNA	XIST decreased During the chondrogenic differentiation of SMSCs from TMJ. XIST knockdown promoted the chondrogenic differentiation of SMSCs. XIST directly bound to miR-27b-3p and regulated the expression of ADAMTS-5.
[36]	MCCs	MCCs from mice were induced by IL-1β (A TMJ-OA model in vitro).	miRNA	*MMP13*, miR-140-5p, and NF-kB were significantly increased in IL-1β inflammatory responses in MCCs. miR-140-5p regulates TMJ-OA pathogenesis through TGF-β/Smad.
[37]	TMJ tissues	TMJ rabbits with surgically induced AL. TMJ from rats with MIA-induced OA.	DNA methylation	The overexpression of Dnmt3b in TMJ stem/progenitor cells led to elevated levels of collagen type II and a reduction in collagen type X, while Dnmt3b knockdown produced the reverse pattern—diminished expression of collagen type II alongside an increase in collagen type X.

ADAMTS-5: A disintegrin and metalloproteinase with thrombospondin motif 5; BMSCs: bone marrow mesenchymal stem cells; circRNA: circular RNA; Dnmt3b: DNA methyltransferase 3 beta; lncRNA: long non-coding RNA; MARK4: microtubule affinity-regulating kinase 4; MCCs: primary mandibular condylar chondrocytes; miRNA: micro RNA; MMP: matrix metallopeptidase; NF-κB: nuclear factor κB; SAHA: histone deacetylase inhibitor suberoylanilide hydroxamic acid; SMSCs: synovium-derived mesenchymal stem cells; TGF-β: transforming growth factor-beta; TMJ-OA: temporomandibular joint osteoarthritis; UAC: unilateral anterior crossbite; XIST: LncRNA X-in- active-specific transcript.

**Table 2:** Comparative Table: TMJ-OA vs. knee/hip OA.

Feature	Temporomandibular Joint Osteoarthritis (TMJ-OA)	Knee/Hip Osteoarthritis (OA)	References
Joint Structure	Synovial joint with fibrocartilage lining	Synovial joints with hyaline cartilage	[3,9]
Primary Function	Mastication, speech; low-load, high-precision	Load-bearing (locomotion and posture)	[121]
Mechanical Stress Type	Shear and compressive (chewing)	Predominantly compressive and torsional	[12]
Cartilage Regenerative Capacity	Higher due to fibrocartilage	Lower hyaline cartilage is poorly regenerative	[3]
Common Age Group	Often affects younger adults (20–40s), especially females	Typically affects older adults (50+ years)	[2]
Symptoms	Jaw pain, joint sounds (clicking/crepitus), and limited motion	Pain, stiffness, swelling, and decreased mobility	[1]
Imaging Findings	Condylar erosion, osteophytes, and joint space narrowing (MRI/CBCT)	Joint space narrowing, osteophytes, and subchondral sclerosis (X-ray/MRI)	[122,123]
Inflammatory Profile	Inflammation may be more prominent in the TMJ	Chronic low-grade inflammation typical	[9]
Epigenetic Regulation	TMJ-specific: altered DNA methylation (e.g., *DNMT3B*, *ADAMTS*, *TGF-β*, and *Wnt/β-catenin*); distinct miRNA and lncRNA expression (e.g., miR-140-5p, HOTAIR, and XIST)	Knee/hip: methylation of *SOX9*, *RUNX2*, and *COL2A1*; miR-140 downregulation; and global DNA methylation alterations	[25,119,124]
Therapeutic Research	Focus on epigenetic therapies, e.g., HDAC inhibitors (SAHA and TSA)	Focus on biologicals (e.g., anti-NGF) and joint replacements in advanced cases	[32,51]

*ADAMTS*: A disintegrin and metalloproteinase with thrombospondin motifs; CBCT: cone beam computed tomography; *DNMT3B*: DNA (cytosine-5) methyltransferase 3 beta; HOTAIR: HOX transcript antisense intergenic RNA; lncRNA: long non-coding RNA; miR-140-5p: microRNA-140-5p; miRNA: microRNA; MRI: magnetic resonance imaging; *TGF-β*: transforming growth factor beta; *Wnt/β-catenin*: Wnt signaling pathway/beta-catenin pathway.

## Supplementary Materials

The following supporting information can be downloaded at: https://www.mdpi.com/article/10.3390/ijms26083668/s1.
