# Supplementary material for: Epigenetic Biomarkers in Temporomandibular Joint Osteoarthritis: An Emerging Target in Treatment
Source: Int J Mol Sci. 2025 Apr 12;26(8):3668. doi: 10.3390/ijms26083668 (PMC12027526; doi:10.3390/ijms26083668)
Supplement: Supplementary file 1 [file ijms-26-03668-s001.zip › ijms-3569252-supplementary.pdf]

International Journal of *Molecular Sciences*

MDPI

Table S1. Summary of methodological quality and sample details of included studies.

References	Study Sample	Level of Evidence
[29]	Not applicable	Review article summarizing biochemical mechanisms of histone acetyltransferases.
[30]	Not applicable	Review article discussing histone acetylation and transcription.
[31]	Not applicable	Review article on p300/CBP-mediated protein acetylation.
[32]	In vitro experimental study	Human synovium-derived mesenchymal stem cells from temporomandibular joint.
[33]	In vitro experimental study	Human OA cartilage
[34]	In vitro experimental study	Synovium-derived MSCs from TMJ-OA patients
[35]	In vitro experimental study	Synovium-derived MSCs from TMJ
[36]	Not applicable	Review
[37]	In vitro and animal study	Rat TMJ tissue, ATDC5 cells
[38]	Not applicable	Review article on HDACs and their inhibitors in cancer therapy.
[39]	Not applicable	Review article examining HDAC inhibitors in various diseases.
[40]	In vitro experimental study	Human articular chondrocytes.
[41]	Animal study	Mouse model of collagen antibody-induced arthritis.
[42]	Animal study	Rat model of osteoarthritis.
[43]	In vitro and animal study	Rat chondrocytes and rat model of osteoarthritis.
[44]	In vitro experimental study	Human chondrocytes.
[45]	In vitro experimental study	Human osteoarthritic chondrocytes.
[46]	Observational study	Human knee cartilage samples from osteoarthritis patients and healthy donors.
[47]	In vitro and animal study	Human synovium-derived mesenchymal stem cells and rat model of temporomandibular joint arthritis.
[48]	In vitro experimental study	Rat articular chondrocytes.
[49]	In vitro experimental study	Human synovial fluid mesenchymal stem cells from temporomandibular joint.
[50]	Not applicable	Review article discussing roles of IL-1 and TGF-β1 in osteoarthritis.
[51]	In vitro experimental study	Human OA chondrocytes
[52]	Not applicable	Review
[53]	In silico study	Computational analysis
[68]	Not applicable	Review
[69]	In vitro experimental study	Tumor cell lines
[70]	In vitro experimental study	Human TMJ-OA fibroblasts
[71]	Clinical study (cross-sectional)	OA patient plasma
[72]	In vitro experimental study	Human monocytic cells
[73]	In vitro experimental study	Human OA chondrocytes
[74]	In vitro experimental study	Human chondrocytes
[75]	Not applicable	Review
[76]	Not applicable	Review
[77]	In vitro and animal study	OA patient tissues & mouse model
[78]	In vitro experimental study	Human TMJ samples
[79]	In vivo study	Rat arthritis model
[80]	In vitro experimental study	Human OA synoviocytes
[81]	In vitro experimental study	Human OA chondrocytes
[82]	In vitro experimental study	Human OA chondrocytes

[83]	Not applicable	Review
[84]	Not applicable	Review
[85]	In vitro and animal study	FLS-derived exosomes, OA rat model
[86]	Not applicable	Review
[87]	Clinical commentary	TMJ arthritis patients
[88]	In vitro experimental study	Human chondrocytes
[89]	In vitro experimental study	Human OA chondrocytes
[90]	In vitro experimental study	Human OA chondrocytes
[91]	In vitro experimental study	Human synoviocytes & chondrocytes
[92]	Not applicable	Review
[93]	Not applicable	Review
[94]	Human cancer data	Meta-analysis
[95]	In vitro and animal study	Human cartilage explants, mouse model
[96]	In vitro experimental study	Human OA chondrocytes
[97]	In vitro experimental study	Human OA chondrocytes
[98]	In vitro and animal study	Human OA chondrocytes, DMM mouse model
[99]	UK Biobank cohort (thousands of OA patients & controls)	Human genetic association study
[100]	In vitro and animal study	Human chondrocytes, OA rat model
[101]	In vitro and animal study	Human TMJ chondrocytes and synoviocytes, mouse model
[102]	Not applicable	Review
[103]	Genome-wide methylation study	Human OA chondrocytes
[104]	Gene expression & methylation profiling	Human OA hip cartilage
[105]	DNA methylation profiling	Human OA cartilage
[106]	Not applicable	Review
[107]	Not applicable	Review
[108]	Animal study	Mouse bone tissue
[109]	Not applicable	Review
[110]	Not applicable	Review
[111]	Animal study	β-catenin conditional activation mice
[112]	Animal study	Chondrocyte-specific β-catenin activated mice
[113]	Animal study	Hematopoietic stem cells from mice
[114]	Not applicable	Review

ATDC5 cells: Mouse chondrogenic cell line; DMM: Destabilization of the medial meniscus; FLS: Fibroblast-like synoviocytes; HDACs: Histone deacetylases; OA: Osteoarthritis; TMJ: Temporomandibular joint; IL-1: Interleukin-1; MSC(s): Mesenchymal stem cells; TGF-β1: Transforming growth factor beta 1; TMJ-OA: Temporomandibular joint osteoarthritis.
